# Magnitude of visual impairment and associated factors among patients attending ophthalmic clinics of Debre Markos referral hospital, north West Ethiopia

**DOI:** 10.1186/s12886-021-01863-0

**Published:** 2021-02-19

**Authors:** Haimanot Abebe, Fasil Wagnew, Haymanot Zeleke, Bitew Tefera, Shegaw Tesfa, Tamene Fetene

**Affiliations:** 1 Department of Nursing, Wolkite University, Wolkite, Ethiopia; 2 Department of Nursing, Debre Markos University, Debre Markos, Ethiopia

## Abstract

**Background:**

Globally, visual impairment affects about 285 million (4.25%) people, of those, 266.4 million were adults aged 18 years and above. Ethiopia is one of developing countries estimated to have high prevalence of visual impairment which have an enormous socio-economic impact. Also there is limited available information regarding with the magnitude of visual impairment among adults in our country at large and east Gojjam zone in specific. Therefore the aim of this study was to assess the magnitude of visual impairment and its associated factors among patients attending Debre Markos Referral Hospital ophthalmic clinics in east Gojjam zone, North West Ethiopia.

**Methods:**

An institutional-based cross-sectional study was conducted at Debre Markos Referral Hospital which is the only hospital in east gojjam zone with ophthalmic care service from March 1 to 30, 2020 by using systematic random sampling technique to select study participants after informed consent was obtained.

Data were collected by interview with 5% pretested, structured questionnaire and ocular examinations. Data were cleaned, coded and entered to Epi-data version-3.1, and analyzed using Statistical Package for Social Science software version 26. The descriptive statistics was presented in tables, text and graphs. Bivariable and multivariable logistic regression analysis to identify factors associated with visual impairment was conducted. Covariates with *P*-value < 0.05 were considered statistically significant.

**Results:**

A study was conducted among 312 study participants with 96% response rate. The magnitude of visual impairment was 114 (36.5%) [95% CI, (33.8, 39.2%)]. Age > 50 years [AOR = 3.82; 95% CI (1.56, 9.35)], rural residency [AOR = 4.33 95% CI (1.30, 14.44)], inability to read and write [AOR = 3.21; 95% CI (1.18, 8.73)] and Cataract [AOR = 4.48; 95% CI (1.91, 10.52)] were factors significantly associated with visual impairment.

**Conclusions:**

The overall magnitude of visual impairment was found to be high. Older age, rural residency, inability to read and write and cataract were associated with visual impairment. Increasing literacy, expanded cataract surgery, as well as community based visual acuity screening especially for elders and rural residents is crucial. Zonal police makers should give emphasis on prevention of visual impairment to decrease economic, social and political burden of visual disability.

**Supplementary Information:**

The online version contains supplementary material available at 10.1186/s12886-021-01863-0.

## Background

Visual system is one of our most important sensory systems which are an essential means of integration between individual and the external environment. Vision is the entrance of light into the eyes and interpretation of this stimulus by the brain and visual acuity is the ability of the eye to see and distinguish fine details [1].

Vision is very important to perform daily physical activities and to lead functional social life [1]. Visual impairment refers to a functional limitation of the eye or visual system due to a disorder or disease that result in poor vision in either or both eyes. Based on World Health Organization revised definition, it is defined as presenting distance visual acuity worse than 6/18 in the better eye whereas presenting distance visual acuity (PVA) in the better eye of < 3/60 is classified as blindness [2]. Snellen “E” chart is usually used to measure distant visual acuity at a distance of 6 m and visual acuity should be recorded as smallest line in which individual being tested can identify the four letters correctly [3].

The number of people affected by visual impairment has increased substantially as the population increases and ages [4]. In Ethiopia all major factors of low vision and blindness are either preventable or treatable [5, 6]. Globally in 2015, an estimated 36.0 million were blind, 216.6 million people had moderate to severe visual impairment, and 188·5 million had mild visual impairment [7]. Even if considerable effort has been made in developing countries; global prevalence of visual impairment seems to be growing mainly as an effect of increasing population and aging [4, 7]. Similarly in Ethiopia, it is still a major public health problem with high prevalence of visual impairment [blindness (1.6) and low vision (3.7)] [5].

Visual impairment leads to a variety of public health, social, and economic problems, notably in developing countries where over 90% of world’s individuals with visual impairment live [4]. It leads to restriction in all areas of life and in particular vision related quality of life [8]. High burden of eye diseases in Ethiopia is believed to pose huge economic and social impacts on individuals, society and the nation at large [5]. Impact is also for care givers like children unable to reach school and adults to stop working [9].

Visual impairment profoundly influences quality of life indicated by more depressive symptoms, high risk of fall, disabilities and lower life satisfaction among older adults [5, 8]. In addition, greater VI significantly predicts increases in activity limitations, financial strain, declines in social integration and self-efficacy which results in loss of productivity and dependency [5, 9–11].

Previous studies conducted globally and in different European and African countries including Ethiopia showed that being older age [12–22], rural residency [16, 23], lower educational status [14–16, 23, 24], low monthly income [15, 16, 23, 25], cataract [4, 12, 13, 20–22, 24, 26], glaucoma [4, 13, 21, 22, 26], macular degeneration [4–6, 17, 22, 26–28], chronic comorbid illnesses [12, 15, 20, 26, 29–31] and smoking [15, 32] were factors associated with unilateral or bilateral visual impairment.

In most sub-Saharan African countries including Ethiopia visual impairment is a result of either preventable or treatable morbidities due to a combination of factors such as lack of education, poverty, inadequate health-care services and material resource [5, 33, 34].

In Ethiopia regardless of multipronged approaches and strategies to reduce the burden of visual impairment through promoting use of eye glasses, increasing public awareness, free mass campaign service on cataract and trichiasis surgery, blinding factors are still on the rise due to growing population and aging [34].

Most studies conducted in Ethiopia were focused on childhood visual impairment giving less attention to adulthood visual impairment [35, 36], some studies were conducted as retrospective chart review which lacks some variables like family history of eye disease, history of eye trauma, chronic co morbidity and life style factors [21, 22] and others were community surveys that are outdated and lacks clinical variables of visual impairment [5]. In Ethiopia, there are also newly emerging factors of visual impairment associated with chronic diseases and advancement in age. Therefore there is a need to identify a risk factors and assess the prevalence of visual impairment for the establishment of zonal and national prevention strategies [7].

## Methods

### Study design, setting and sampling

Institutional based cross sectional study was conducted from March 1 to 30 /2020 in Debre Markos Referral Hospital /DMRH/ which is the only hospital having ophthalmic clinic in East Gojjam zone of Amhara Regional State, 300 km north of Addis Ababa, the capital city of Ethiopia and 265 KM away from Bahir Dar the city of Amhara region with a catchment population of 3.8 million people.

Clients who were 18 years of age and above visiting DMRH eye clinic during the data collection period were included in the study.

The minimum required sample size for the first objective was obtained by using a single population proportion formula by taking the prevalence of visual impairment (15.3%)conducted in Gondar teaching hospital in 2012 [21]. at 95% CI by assuming a margin of error 5% =0.05) and adding 15% for possible non response rate which was 230. For second objective, double population proportion formula was used for sample size determination by using Epi-info 7 software at 95% CI with 5% margin of error, 90% power, 1:1 ratio of exposed to non-exposed outcomes and adding 15% for possible non response rate based on the study conducted in Debre Berhan Town, North Shewa, Ethiopia 2018 and taking the maximum value the final sample was 325. Systematic random sampling technique based on patient arrival was used to recruit the study participants every K value by selecting the first respondent using lottery method. Debre Markos Referral Hospital provides eye care service for approximately 40–75 patients, in average of around 58 patients per day in all working days from Monday to Friday and those it is estimated that it serves approximately 1276 patients in the last year similar month with this study data collection period. With this K value was calculated as 1276/325 = 4 and systematic random sampling technique based on patient arrival was used to recruit the study participants every K value by selecting the first respondent using lottery method.

### Operational definitions

Visual impairment was defined according to the revised World Health Organization international Classification of Disease (ICD)-10; 2016 definitions of VI criteria [2].

1. Normal vision:- A presenting VA of > 6/18 in the better eye
2. Visual impairment: - A visual acuity of less than 6/18 in the better eye.
  - Moderate visual impairment (PVA < 6/18–6/60)
  - Sever visual impairment (PVA < 6/60–3/60)
  - Blindness: - A visual acuity of less than 3/60 in the better eye
  - Low vision: - Presenting visual acuity 6/18 but not less than 3/60 in better eye.

### Data collection tool and procedure

Pre-tested (5%) structured questionnaire developed after the review of different literature was used to collect data. The questionnaire was containing 4 parts: socio-demographic assessing questions, Personal and life style assessing questions, clinical variables and chronic co-morbidity assessing questions. Two clinical nurses, two optometrist, four ophthalmic nurses and one ophthalmologist as data collectors and one BSc nurse as a supervisor all working at Debre Markos referral hospital were involved in the data collection process after 3 day training by the principal investigator.

Socio-demographic data: sex, age, level of education, occupation, marital status, and residence and other relevant information related to visual impairment, such as watching TV, playing computer game, using eye glass, smoking cigarrete, alcohol use, previous ophthalmic clinic visit, chronic comorbidity, history of eye trauma, family history, previous vision problem and history of eye disease were collected by clinical nurses through face to face interview and chart review.

Clinical nurses performed visual acuity test for each eye using the Snellen chart at a distance of 6 m. Snellen “E” chart had a capital letter “E” turning in different directions either upward, down ward, left or right and the client being tested should determine which direction “E” is facing at a distance of 6 m. Presenting visual acuity was recorded as the smallest line in which the client can identify and read the four letters correctly. If the client was unable to identify the largest letter “E” at a distance of 20 ft / 6 m, finger counting was done initially at a distance of 1 m and gradually increasing the distance until the client no longer identify the examiner fingers. If the client was unable to count the fingers at a distance of 1 m but see the examiners hand moving, “hand motion” was recorded as a visual acuity. If he/she cannot identify hand motion, Torch was held in front of the participant’s eye and asked to tell when torch light is on. If he/she can correctly identify when the torch light is on, “light perception” was recorded, if not “NLP/no light perception” was recorded. Care was taken to ensure that the unexamined eye was adequately covered using cards. For those with PVA of < 6/18 optometrists were performed pinhole test and subjective refraction for refractory error using standard refraction trial set. Vision impairment was attributed to refractive error if PVA improved to > 6/18 with refraction.

Ophthalmic nurses were performed external eye examination by using torch light for corneal opacity test and to identify external eye diseases, slit lamp examination to examine external eye structures, lens, iris and anterior chamber, ophthalmoscopy to examine the retina, vitreous body and macule as well as tonometry for measuring intra ocular pressure (IOP). Ocular abnormalities for some patients which could not been identified by ophthalmic nurses were identified by a senior ophthalmologist.

### Data quality control

Data were collected using structured questionnaire; 5% Pre-test was done at Felege Hiwot Referral Hospital, Amhara region, Ethiopia and necessary correction on vague questions was done before actual data collection. Data were entered directly in to Epi-data software and cleaned and checked for completeness and accuracy before analysis. The data collection instrument was validated through pre-test and clinical expert’s evaluation,

All data collectors and supervisor were provided 3 day training for mutual consensus about data collection tool and data collection process. Unclear or ambiguity issues during data collection procedure were resolved by a principal investigator. Over all cronbach’s alpha value was used to check the reliability of the tool which accounted for 0.83 and its quality was assured through experts’ evaluation. During the data collection period, 5% of the data were cross checked daily for completeness by principal investigator.

### Data analysis

After data were checked for completeness and consistency, it was coded and entered in to EPI data version 3.1 and exported to SPSS version 26 for analyses. Descriptive statistics was computed and presented using text, frequency tables, graphs, percentage, mean and standard deviation.

Binary logistic regression model in enter method was used for analysis, and variables with *p*-value < 0.25 in bivariable logistic regression was fitted to multivariable logistic regression. The strength of association of a particular variable was expressed as adjusted odds ratio (AOR) with a 95% confidence interval. A two-tailed t-test *P* value of < 0.05 was declared as a statistically significant. Moreover, variance inflation factor (VIF) and tolerance to check for multicollinarity and Hosmer and Lemeshow goodness of fit test to check for model fitness was used (it was 0.936).

## Result

### Socio-demographic characteristics

The study was conducted among 312 participants attending DMRH eye clinic during the data collection period with a response rate of 96% of which 193 (61.9%) were males and the mean age of respondents were 46.17 (+ 18.95 SD). Almost three fourth of the respondents were married 227 (72.8%), majority of them were orthodox religion followers 297 (95.2) and above half of the participants were rural dwellers 180 (57.7%) (Table 1).

**Table 1:** Socio-demographic characteristics of the study participants, DMRH eye clinic (*N* = 325); March 2020

Socio-demographic variable	Category	Frequency	Percentage (%)
Sex	Male	193	61.9
	Female	119	38.1
Age	< 40	126	40.4
	40–49	34	10.9
	50 and above	152	48.7
Marital status	Married	227	72.8
	Not married	85	27.2
Residence	Urban	132	42.3
	Rural	180	57.7
Religion	Orthodox	297	95.2
	Muslim	15	4.8
Ethnicity	Amhara	310	99.4
	Oromo	2	.6
Educational status	Can’t read and write	136	43.6
	Can read and write	40	12.8
	Primary school and above	136	43.6
Occupation	Employed	69	22.1
	Unemployed	243	77.9
Family size	< 5	143	45.8
	5 and above	169	54.2
Health insurance	Yes No	205 107	65.7 34.3

### Personal and life style factors

Among 312 respondents, above half 199 (63.8%) of them did not watch TV and more than three-fourth of participants 249 (79.8%) having no mobile cellphone and computer game Exposur*e. major*ity of the participants 198 (63.5%) ever drink alcohol but no any history of smoking cigarette 302 (96.8%) (Table 2).

**Table 2:** Personal and life style characteristics of the study participants at Debre Markos Referal Hospital eye clinic (*n* = 312); March 2020

Personal and life style factors	Category	Frequency	Percentage (%)
Watching TV at least once/day	Yes	113	36.2
	No	199	63.8
Frequency of TV exposure per day	< 2 h.	53	46.9
	2–4 h.	51	45.1
	> 4 h	9	8.0
Watching TV distance	< 2 m	1	0.9
	2–4 m	95	84.1
	> 4 m	17	15.0
Mobile phone or computer game exposure	Yes	63	20.2
	No	249	79.8
Mobile phone or computer game exposure/day	< 2 h.	15	23.8
	2–4 h.	33	52.4
	> 4 h.	15	23.8
Wearing eyeglass	Yes	39	12.5
	No	273	87.5
Eye glass type	Reading	15	38.5
	Distant	6	15.4
	Photochromic	18	46.1
How often do you wear eyeglass?	Always	6	15.4
	Usually	11	28.2
	Sometimes	22	56.4
Ever smoke cigarette	Yes	10	3.2
	No	302	96.8
Ever drink alcohol	Yes	198	63.5
	No	114	36.5

### Previous medical history and comorbidity factors

Most of the study participants had no any family history of vision problem 273 (76%). Whereas, almost two-thirds 210 (67.3) of respondents had previous history of ophthalmic clinic visit, 37 (11.9%) had hypertensive disease and 26 (8.3%) had history of tuberculosis of whom all were taking anti-tuberculosis medication (Table 3).

**Table 3:** Previous medical history and clinical comorbidity characteristics of the study participants at DMRH eye clinic; March 2020 (*N* = 312)

Previous history and comorbidity	Category	Frequency	Percentage (%)
Family history of vision problem	Yes	75	24.0
	No	237	76.0
Previous ophthalmic clinic visit	Yes	210	67.3
	No	102	32.7
Hypertension /HTN/	Yes	37	11.9
	No	275	88.1
Anti-hypertensive medication	Yes	26	70.3
	No	11	29.7
Diabetic mellitus/DM/	Yes	10	3.2
	No	302	96.8
Anti-Diabetic medication	Yes	9	90
	No	1	10
Tuberculosis	Yes	26	8.3
	No	286	91.7
Tuberculosis treatment	Yes	26	100.0
HIV/AIDS	Yes	8	2.6
	No	304	97.4
HAART	Yes	8	100.0
History of ocular trauma	Yes	63	20.2
	No	249	79.8
History of vision problem	Yes	86	27.6
	No	226	72.4
Other chronic diseases (CHD, Asthma, CKD, Cancer)	Yes	14	4.5
	No	298	95.5

### Clinical factors of study participants

Among the study participants, the leading clinical diagnosis was glaucoma 72 (23.1%) followed by cataract 65 (20.8%) and allergic conjunctivitis 60 (19.2%) (Table 4).

**Table 4:** Frequency distribution of clinical factors among participants at Debre Markos Referral Hospital eye clinic; March 2020 (*N* = 312)

Clinical factor	Category	Frequency	Percentage (%)
Cataract	Yes	65	20.8
	No	247	79.2
Glaucoma	Yes	72	23.1
	No	240	76.9
Uncorrected refractive error (URE)	Yes	32	10.3
	No	280	89.7
Corneal opacity (CO)	Yes	18	5.8
	No	294	94.2
Retinopathy	Yes	11	3.5
	No	301	96.5
Trachoma	Yes	12	3.8
	No	300	96.2
Bacterial conjunctivitis	Yes	20	6.4
	No	292	93.6
Unspecified disease	Yes	8	2.6
	No	304	97.4
Others diseases	Yes	174	55.8
	No	138	44.2
Magnitude of diseases under other diseases classification			
Macular degeneration	Yes	13	4.2
	No	299	95.8
Retinal detachment (RD)	Yes	8	2.6
	No	304	97.4
Ocular trauma (OT)	Yes	20	6.4
	No	292	93.6
Allergic conjunctivitis	Yes	60	19.2
	No	252	80.8
Others	Yes	93	29.8
	No	219	70.2

### Magnitude of visual impairment

**Fig. 1:**
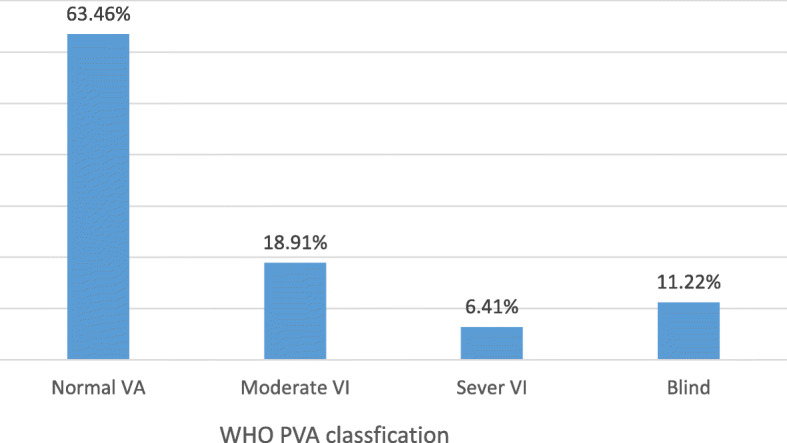
Magnitude of VI based on WHO visual impairment severity classification at DMRH ophthalmic clinic; North West Ethiopia; March 2020 (*N* = 312)

Among a total of 312 study participants 114 (36.5%) had visual impairment of whom 35 (11.2%) were blind. In this study the prevalence of low vision and unilateral visual impairment was 25.3% and 82 (26.3%) respectively. The most prevalent eye diseases in people with visual impairment were cataract 48 (42.1%), glaucoma 42 (36.8%), MD 12 (10.5%), CO 10 (8.8%) and URE 9 (7.9%). Magnitude of visual impairment based on WHO severity classification (Fig. 1).

### Factors associated with visual impairment

In bivariate analysis the covariates: Age, marital status, residence, educational status, family size, health insurance, watching TV, Mobile phone/computer games exposure, wearing eye glasses, drinking alcohol, previous ophthalmic clinic visit, HTN, previous history ocular trauma, previous vision problem, cataract, glaucoma, corneal opacity and diseases classified as others (MD, RD, macular hole, papilledema, pseudophakia, ocular trauma) were associated with visual impairment.

In multivariable analysis, age, residence, educational status and cataract were significantly associated with visual impairment at *P*-value < 0.05 level of significance and 95% confidence interval.

Being age 50 years and above was 3.8 times more risky to have visual impairment as compared to those age < 40 years [AOR = 3.82; 95% CI (1.56, 9.35)]. Rural dwellers were nearly 4 times more likely to have visual impairment as compared to urban residents [AOR = 4.33; 95% CI (1.300, 14.44)].

The odds of visual impairment among those who cannot read and write were 3 times as compared to those who have primary level of education and above [AOR = 3.21; 95% CI (1.19, 8.73)]. Cataract patients were almost 4.5 times more risk for visual impairment than those individuals without cataract [AOR = 4.48; 95% CI (1.91, 10.52)] (See additional file 1).

## Discussion

In this study the magnitude of visual impairment was 36.5% with 95% CI (33.8, 39.2%) which was higher as compared to a study conducted in Saudi Arabia (13.9%) [12], Eastern Taiwan (11.0%) [20], Juaben, Ghana (28.2%) [13], Gondar, Ethiopia (15.3%) [21], Debre Berhan, Ethiopia (16.8%) [14], and St.paul’s hospital; Addis Ababa, Ethiopia (17.6%) [22] .

The discrepancy might be due to population difference, in which Saudi Arabia study participants were those who attend primary health care center for any type of health care service and Eastern Taiwan and Debre Berhan town, Ethiopia studies were community based studies with increased chance of screening normal sighted people as compared to a current hospital based study where participants already came with certain complain. Similarly, a study in Debre Birihan Ethiopia involved only urban residents which underestimate the magnitude of visual impairment related to rural residency as evidenced from this study where being rural dweller were associated with visual impairment due to lack of concept and awareness on health as well as poor health care accessibility.

It might be also due to difference in definition of visual impairment from Saudi Arabia study which uses best corrected visual acuity (BCVA) that can underestimates visual impairment related to uncorrected refractive error. Another possible justification for the discrepancy might be due to research methodology difference from Juaben Hospital eye clinic, Ghana, St.paul’s hospital; Addis Ababa, and Gondar teaching hospital, Ethiopia studies which were used retrospective chart review that might use visual acuity after correction for refractive errors which lowers magnitude of visual impairment related to refractive error. Another reason might be due to participants age difference from eastern Tiwan and St.paul’s hospital; Addis Ababa, Ethiopia studies that includes all age groups and University of Gondar teaching hospital study includes patients’ age 14 years and above which might lower the prevalence of visual impairment related to aging [37]. The discrepancy might be also due to difference in technological advancement, better awareness and better health care facility, in Taiwan, Ghana and Saudi Arabia.

In this study the prevalence of blindness (PVA < 3/60 in the better eye) was 35 (11.2%) with 95% CI (7.8, 14.6%) which was similar with a study conducted at university of Gondar teaching hospital (14.4%) [21]. The reason might be similarity in socio-economic characteristics, study population, study area and similar cut of point (VA < 3/60) for defining blindness. On the other hand, it was higher than a study conducted in Ghana (3.7%) [13], and St.paul’s hospital millennium medical college; Addis Ababa, Ethiopia (7.3%) [22] which might be due to research method and age difference, others were a retrospective studies and include all age groups that lower the magnitude of blindness due to age related visual disabling morbidities like macular degeneration and atrophy [38] in contrast to a current study which includes those 18 years of age and above as well as difference in technology and geographical area from Ghana .

In this study age > 50 years, rural residence, inability to read and write and cataract were significantly associated with visual impairment. Age 50 and above years were positively associated with visual impairment which was supported by different Global studies [17, 18], a studies conducted in Urban Asian Population [15], Saudi Arabia [12], eastern Taiwan [20], Ghana [13] and Chinese adults [16] and also in line with a study conducted in Ethiopia at university of Gondar teaching hospital [21], Debre Birihan town [14] and St.paul’s hospital millennium medical college; Addis Ababa [22]. The possible reason for increased visual impairment in old age might be due to increasing age related eye diseases and degenerations [3, 38]. As age increases, the function of all body including visual system become less efficient as a result of physiological deterioration as well as increased exposure to ocular infections due to deterioration of the eye structure and people may suffer more and more eye diseases related to aging such as macular degeneration, cataract, retinopathy and retinal dystrophy, which leads to visual impairment [38].

The odds of visual impairment in rural dwellers were nearly 4 times higher than urban residents. This finding was in line with a study conducted in Korea [23] and Chinese adults [16]. This might be due to lack of awareness on health and preventable factors of visual impairment, inadequate health care accessibility and infrastructures, far distance for traveling to ophthalmic clinics in rural areas, inequality of access to eye care services concentrating at urban areas. Low living standard, less educated, poor environmental and personal sanitation practice in rural areas leading to increased prevalence of ocular infection and associated visual impairment as compared to urban areas [39]. Rural residents may not seek medical attention for early detection and treatment of eye problems due to ignorance and lack of concept on health, usually seeking eye care lately after visual disability that prevents them from working [33, 39] .

In this study, being unable to read and write was associated with visual impairment which was supported by other studies done in Urban Asian Population [15], India [24], in Korea [23], China [16], and Debre Birihan, Ethiopia [14]. The possible reason might be due to their poor health related behaviors and lack of awareness on preventive mechanisms of visual impairment. Those who can’t read and write may have poor habit of utilizing available eye care services [40]. Uneducated individuals may also had lesser awareness due to challenge on reading printed public messages on health, unaffordability and poor access to eye care services compared to educated individuals having high visual demand usually concentrating at urban areas with better facility [33, 40].

In the current study cataract patients were high risk of having visual impairment as compared to those without cataract which were similar with a study conducted in Eastern Taiwan [20], Saudi Arabia [12], India [24], Ghana [13], St.paul’s hospital millennium medical college; Addis Ababa, Ethiopia [22] and university of Gondar teaching hospital [21] which is due to progressive nature of the disease that causes lens opacity obliterating the focus of light into the retina where image is created. It usually causes painless and progressive blurring of vision that a patient may not seek medical attention until visual impairment and prevents them from accomplishing their daily activity of living [3, 41] and might be due to long waiting time for cataract surgery until cataract maturity leading to impaired vision.

Even if early diagnosis and prompt surgery consisting of removing affected lens and replacing it with artificial one is the only management option for cataract which has no proven medical or preventive therapy patients usually seek eye care lately after visual disability due to fear of surgical complication and misconceptions about eye procedures like cataract surgery and its benefit especially in rural dwellers that they might totally avoid it and decreased utility of available ophthalmic clinics leading to progressive visual impairment and disability later on [41].

## Conclusion

The magnitude of visual impairment in this study was found to be high as compared to previous studies. Older age, rural dweller, inability to read and write and cataract were significantly associated with visual impairment. Zonal police makers should give emphasis on prevention of visual impairment to decrease economic, social and political burden of visual disability.

### Limitation of the study

It was a cross-sectional study that might not show cause and effect relationship. Since it was a hospital based study conclusion on the prevalence of visual impairment in the community cannot be made.

## Supplementary Information

**Additional file 1.** Multivariable logistic regression analysis of factors associated with visual impairment at DMRH ophthalmic clinic; northwest Ethiopia, March, 2020.

## Data Availability

The datasets used and/or analyzed during the current study available from the correspondingauthor on reasonable request.

## Abbreviations

AOR: Adjusted Odds Ratio
CI: Confidence Interval
COR: Crude Odds Ratio
DMRH: Debre Markos Referral Hospital
EPI INFO: Epidemiological Information
NLP: No Light Perception
MMD: Myopic Macular Degeneration
PVA: Presenting Visual Acuity
SPSS: Statistical Package for Social Science
VI: Visual Impairment
WHO: World Health Organization
