# Supplementary material for: Magnitude of visual impairment and associated factors among patients attending ophthalmic clinics of Debre Markos referral hospital, north West Ethiopia
Source: BMC Ophthalmol. 2021 Feb 19;21:96. doi: 10.1186/s12886-021-01863-0 (PMC7893842; doi:10.1186/s12886-021-01863-0)
Supplement: Supplementary file 1 — Additional file 1. Multivariable logistic regression analysis of factors associated with visual impairment at DMRH ophthalmic clinic; northwest Ethiopia, March, 2020. [file 12886_2021_1863_MOESM1_ESM.pdf]

Additional file 1: Multivariable logistic regression analysis of factors associated with visual impairment at DMRH ophthalmic clinic; northwest Ethiopia, March, 2020

Explanatory Variable	Category	Visual impairment		COR (95% CI)	AOR (95% CI)	P-value
		YES	NO			
Age	<40	14	112	1.00	1.00	
	40-49	10	24	3.33(1.32, 8.39)	1.46 (0.48, 4.44)	0.510
	≥50	90	62	11.6(6.11, 22.1)	3.82 (1.56, 9.35)	0.003*
Residence	Urban	28	104	1.00	1.00	
	Rural	86	94	3.39(2.04, 5.66)	4.33 (1.30, 14.44)	0.017*
Marital status	Married	105	122	1.00	1.00	
	Not married	9	76	0.14(0.07, 0.29)	0.49 (0.19, 1.26)	0.139
Educational status	Can't read and write	78	59	8.0(4.43, 14.46)	3.21 (1.18, 8.73)	0.023*
	Can read and write	17	24	4.29(1.95, 9.43)	1.55 (0.53, 4.55)	0.426
	Primary education and above	19	115	1.00	1.00	
Family size	< 5	46	97	1.00	1.00	
	≥ 5	68	101	1.42(0.89, 2.26)	0.76 (0.39, 1.47)	0.410
Health insurance	Yes	87	118	1.00	1.00	
	No	27	80	0.46(0.27, 0.77)	1.44 (0.69, 3.00)	0.328
Watching TV	Yes	26	87	0.38(0.22,0.63)	1.75 (0.48, 6.39)	0.397
	No	87	111	1.00	1.00	
Mobile/computer game	Yes	9	54	0.23(0.11, 0.48)	1.39 (0.47, 4.08)	0.550
	No	105	144	1.00	1.00	
Eye glass	Yes	11	28	1.00	1.00	
	No	103	170	1.54(0.74, 3.23)	0.88 (0.32, 2.41)	0.803
Alcohol	Yes	79	119	1.49(0.92, 2.44)	0.68 (0.32, 1.43)	0.308
	No	35	79	1.00	1.00	

Additional file 1: Multivariable logistic regression analysis of factors associated with visual impairment at DMRH ophthalmic clinic; northwest Ethiopia, March, 2020

Previous visit	Yes	87	123	1.00	1.00	
	No	27	75	0.51(0.30, 0.86)	0.48 (0.23, 1.00)	0.050
HTN	Yes	20	17	2.27(1.13, 4.53)	2.14 (0.83, 5.54)	0.117
	No	94	181	1.00	1.00	
History of ocular trauma	Yes	16	47	0.52(0.28, 0.98)	0.51 (0.23, 1.14)	0.099
	No	98	151	1.00	1.00	
Previous vision problem	Yes	38	48	1.56(0.94, 2.59)	0.98 (0.50, 1.89)	0.941
	No	76	150	1.00	1.00	
Cataract	Yes	48	17	7.74(4.16, 14.4)	4.48 (1.91, 10.52)	0.001*
	No	66	181	1.00	1.00	
Glaucoma	Yes	42	30	3.27(1.89, 5.63)	2.06 (0.87, 4.89)	0.100
	No	72	168	1.00	1.00	
Corneal opacity	Yes	10	8	2.28(0.87, 5.96)	2.59 (0.72, 9.41)	0.146
	No	104	190	1.00	1.00	
Other diseases	Yes	47	127	0.39(0.24, 0.63)	1.37 (0.68, 2.79)	0.382
	No	67	71	1.00	1.00	

''*'' for variables significantly associated with visual impairment at P-value < 0.05
Other diseases (MD, RD, papilledema, Pseudophakia, ocular trauma...)
